# Transnational Caregiving for Older Adults and Caregivers’ Wellness in Japanese Americans during the Pandemic

**DOI:** 10.1093/geroni/igab046.3542

**Published:** 2021-12-17

**Authors:** Kazumi Hoshino, Winston Tseng, Kei Kamide

**Affiliations:** 1 The University of California at Berkeley, Berkeley, California, United States; 2 Osaka University, Suita, Osaka, Japan

## Abstract

Global migration has greatly affected intergenerational family support beyond national borders, in particular adult children’s transnational family caregiving for elderly parents. Specifically, the COVID-19 pandemic has largely influenced transnational caregiving due to the travel restrictions. Transnational caregiving for older adults includes adult children’s periodical returning to their home country and/or adult children’s caregiving for their parents in their settled country. The goal of this study was to identify trajectories between adult children’s transnational caregiving for their parents and caregivers’ wellness in Japanese Americans before and during the pandemic. We conducted semi-structured interviews with Japanese Americans 40 to 59 years of age (N=20) in California before the lockdown and during the increasing number of patients infected with the Delta variant. The qualitative data analysis showed some Japanese Americans periodically returned to Japan to provide caregiving for their parents before the pandemic, while others didn’t. However, the former group currently relied on their families in their home country more than before. The limitations led to not only distress over uncertainty but also release from a strong sense of reciprocity and filial responsibility, by changing from physical support to emotional and financial support via online. They also enhanced cultural identity as Japanese Americans, by thriving from discrimination against Asian Americans. Thus, our findings demonstrate important factors that impacted on transnational caregiving and caregivers' wellness, including cultural identity, family norms, beliefs and practices of intergenerational support, social and historical contexts, financial remittance, ICT use, and healthcare policies among the underrepresented populations across the Pacific.

